# Effects of Resistant Starch Ingestion on Postprandial Lipemia and Subjective Appetite in Overweight or Obese Subjects

**DOI:** 10.3390/ijerph16203827

**Published:** 2019-10-11

**Authors:** Carlos García-Vázquez, Jorge L. Ble-Castillo, Yolanda Arias-Córdova, Rubén Córdova-Uscanga, Carlos A. Tovilla-Zárate, Isela E. Juárez-Rojop, Viridiana Olvera-Hernández, Carina S. Alvarez-Villagomez, Ana M. Nolasco-Coleman, Juan C. Díaz-Zagoya

**Affiliations:** 1 Centro de Investigación, División Académica de Ciencias de la Salud, Universidad Juárez Autónoma de Tabasco (UJAT), Villahermosa, Tabasco 86150, Mexico; gbasecs@hotmail.com (C.G.-V.);; 2 División Académica Multidisciplinaria de Comalcalco, Universidad Juárez Autónoma de Tabasco, Comalcalco, Tabasco 86650, Mexico; 3 División Académica de Ciencias Biológicas, Universidad Juárez Autónoma de Tabasco. Villahermosa, Tabasco 86150, Mexico; 4 Unidad de Medicina Familiar No. 39, Instituto Mexicano del Seguro Social, Villahermosa, Tabasco 86070, Mexico; 5 División de Investigación, Facultad de Medicina, Universidad Nacional Autónoma de México, Cd. de México 04510, Mexico

**Keywords:** indigestible carbohydrates, resistant starch, postprandial lipemia, appetite, triglycerides

## Abstract

Reports surrounding the role of resistant starch (RS) on postprandial lipemia in humans are scarce. The aim of the present study is to examine the effects of resistant starch on the postprandial lipemic response, subjective measures of appetite, and energy intake in overweight and obese subjects. In a randomized, single-blind, crossover study, 14 overweight/obese participants ate a high-fat breakfast (679 kcal, 58% from fat) and a supplement with native banana starch (NBS), high-amylose maize starch (HMS), or digestible maize starch (DMS) on three separate occasions. All supplements provided were matched by the available carbohydrate content, and the RS quantity in NBS and HMS supplements was identical. Appetite was estimated using visual analogue scale (VAS) and an ad libitum test meal. Postprandial glycemia, triglycerides, cholesterol, high-density lipoprotein (HDL) cholesterol, and insulin excursions did not differ between treatments. Subjective appetite measures of satiety were significantly increased after HMS; however, no effects on energy intake were observed during the ad libitum test meal. These findings suggest that a single acute dose of RS cannot be expected to improve postprandial lipemia in subjects with overweight or obesity on a high-fat meal. However, the potential benefits of long-term supplementation should not be ruled out based on these results.

## 1. Introduction

Typically, the conventional risk factors associated with cardiovascular disease are evaluated in a fasting state. However, in recent years, postprandial dysmetabolism which is characterized by abnormal increases in the circulating levels of glucose and lipids, has been identified as an independent risk factor for the onset of cardiovascular events. In particular, obese individuals develop an increased response in postprandial triglycerides (TG) after the consumption of a fatty meal [1,2]. Acute dietary fiber intake is known to be beneficial for the amelioration of postprandial dysmetabolism; for example, psyllium husk and flaxseeds have been reported to reduce postprandial lipemia in subjects who are overweight and obese [3,4]. Some of the proposed mechanisms are the formation of viscous solutions, delayed gastric emptying, and inhibition of lipase activity [5]. Resistant starch (RS) is considered as a dietary fiber that is resistant to digestion in the small intestine [6], and as such, it is believed to have beneficial effects in reducing postprandial lipemia.

RS is found naturally in various foods and is also available in various industrial preparations. It is classified as type 1, which is inaccessible to digestible enzymes because of certain physical barriers; type 2 RS, which is protected from digestion because of its crystalline structure; type 3 RS, which are retrograded starches formed when starchy foods are cooked and then cooled; type 4 RS, which are chemically modified starches; and type 5 RS, which comprises amylose-lipid complexes and resistant maltodextrin [7]. Unripe bananas are known to be the non-manufactured food with the highest RS content. Native banana starch (NBS) with a high type 2 RS content, is obtained from unripe “Dwarf Cavendish” bananas (Musa, AAA Group) which is widely produced in Tabasco, Mexico. On the other hand, high-amylose maize starch (HMS: Hi-Maize 260^®^) is commercially produced and is the most widely studied type 2 RS. Both of these substances are glucose polymers that are resistant to digestion but differ in their physicochemical properties [8,9].

Most studies about the effects of RS on postprandial lipemia have been conducted in animal models, in which amelioration of the condition has been reported after long-term RS treatment [10,11,12] or acute RS supplementation [13,14]. In humans, studies have mostly focused on the fasting values, based on which it was shown that long-term RS supplementation induces a reduction in total cholesterol and low-density lipoprotein (LDL) cholesterol, but no effects were observed on fasting TG [15]. In contrast, some studies about postprandial lipemia in humans have reported that long-term supplementation of RS has no beneficial effects [16,17,18,19]. Thus, the findings of the published studies are inconsistent; additionally, there is a paucity of studies on the effects of acute supplementation of RS on postprandial lipemia [20,21]. The present study seeks to fill in the gap and contribute to the literature on this topic.

The aim of the present study is to determine the acute effects of RS from the two above mentioned sources (NBS and HMS) on postprandial lipemia, appetite sensations, and caloric intake in subjects with overweight or obesity. We hypothesized that RS supplementation reduces postprandial lipemia and has a positive influence on subjective appetite scores.

## 2. Materials and Methods

### 2.1. Participants

This study was authorized by the Ethics Committee of the Juarez Autonomous University of Tabasco (Universidad Juárez Autónoma de Tabasco (UJAT)), and was conducted in accordance with the tenets of the Declaration of Helsinki and guidelines for the protection of human subjects under research. Participants were recruited through locally advertising in the Health Sciences Department (División Académica de Ciencias de la Salud) of the UJAT. The purpose and potential risks of the study were explained to the volunteers before the written informed consent was obtained from each subject. All participants were notified of the possibility of withdrawing from the experimental protocol at any time they choose. Volunteers did not receive an honorarium to participate in this study.

**Figure 1:**
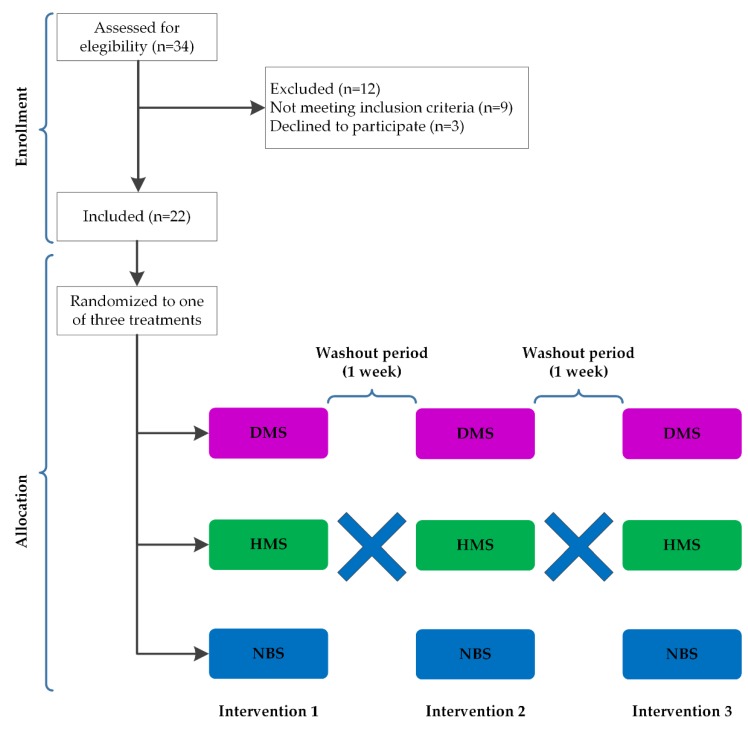
Flow diagram of participants in enrollment, allocation, and experimental phases. A total of 22 volunteers were randomly allocated to receive a high-fat breakfast supplemented with digestible maize starch (DMS), high-amylose maize starch (HMS), or native banana starch (NBS) during one-day intervention. Then, they were crossed over any of the other arms after one-week washout period. The blue crosses represent the crossover design in which the participants cross over from one treatment to another.

A total of 22 overweight or obese participants (BMI ≥ 25 kg/m^2^), between the ages of 18 and 30 years, were included (Figure 1). Those with a history of gastrointestinal disease or alcoholism, gallbladder surgery, cardiovascular events, use of lipid-lowering medication or dietary supplements known to affect appetite, a diagnosis of diabetes or fasting glycemia ≥126 mg/dL, chronic diseases such as liver and renal disorders as well as those that were pregnant or under psychiatric treatment, were excluded. Participants were selected based on a previous medical check-up, anthropometric indexes, and laboratory tests.

### 2.2. Study Design and Protocol

In a randomized, single-blind, crossover design the subjects received native banana starch (NBS), high-amylose maize starch (HMS), or digestible maize starch (DMS) as a supplement. The supplements were randomly assigned, and the participants were not aware of their composition. They were required to report at the laboratory on three separate test days that were at least one week apart. In this way, the participants were switched throughout to all the treatments after a washout period.

Prior to the test days, subjects were advised not to ingest alcohol and avoid vigorous exercise for 24 h. They were also asked to consume standard low-fat and low-fiber evening meals on the three evenings prior to the meal tolerance test (MTT) days. On the day of the treatment, the participants arrived at the research center at 7:00 am after 12 h of fasting and were interviewed to verify that they had adhered to the protocol. Appetite sensation was assessed using a visual analogue scale (VAS; time point 0). Then, an i.v. catheter was inserted into the antecubital vein, and fasting blood sample was obtained. At following, the subjects ate a high-fat breakfast consisting sandwich made of white bread, mayonnaise, Manchego-type cheese, Panela cheese, American cheese, cream cheese, and butter, accompanied with a beverage of semi-skimmed cow’s milk and sucrose. The energy content of the breakfast was 679 kcal distributed as 11 E% protein, 31 E% carbohydrates, and 58 E% fat.

The breakfast was consumed within 15 min after the fasting blood was drawn. Additional blood samples were obtained at the following time points: 30, 60, 90, 120, 150, 180, 240, 300, and 360 min after meal. Also, VAS scores were recorded two minutes prior to each blood sampling. During the course of the test, the subjects remained at the research center and were allowed to read, use their computer, watch television, or talk, as long the conversation did not involve the subject of food. At the end of the 6 h MTT, the catheter was removed and, after a 15 min rest, an ad libitum test meal was provided. This meal consisted of a typical “chicken salad” containing carrots, potatoes, mayonnaise, and chicken. The quantity consumed was calculated by subtracting the portion left on the plate from the portion served. The caloric content per 100 g of salad was 197.5 kcal, corresponding to 12 E% protein, 34 E% carbohydrates, and 54 E% fat. The participants were asked to continue to keep a record of the food they consumed at dinner time. In all cases, the macronutrients and energy content were calculated by a nutritionist according to the Mexican System of Food Equivalents (Sistema Mexicano de Alimentos Equivalentes (SMAE)) [22].

### 2.3. Treatments

The dose of RS (20 g/day) that was chosen in this study was based on the dose reported in previous studies with NBS by our research group [23,24] and with HMS by others [17]. All treatments were matched for the available carbohydrate content (13.3 g), and the NBS and HMS treatments were formulated to contain the same amount of RS (20 g). To prepare the DMS dose, 13.3 g Amioca^®^ (100% rapidly digestible starch) was weighed out. To prepare the HMS dose, 33.3 g of Hi-Maize^®^260 (60% type 2 RS and 40% rapidly digestible starch (RDS)) was weighed, comprising 20 g of RS and 13.3 g of RDS. To prepare the NBS dose, 28.6 g of NBS (70% type 2 RS and 10% RDS) and 10.46 g of Amioca^®^ were mixed. All the doses were dissolved in the beverage that was provided along with the high-fat breakfast.

Hi-Maize^®^ and Amioca^®^ were purchased from Ingredion Mexico S.A. de C.V. (Guadalajara, State of Jalisco, Mexico), and NBS was obtained from unripe (green) bananas (Musa (AAA group)) Dwarf Cavendish (F) with a physiological age of 15 weeks, which were obtained from a fruit packing plant located at 43.5 Km from the Villahermosa-Teapa highway in the Mexican state of Tabasco. NBS was isolated using a previously described procedure with slight modifications [25].

### 2.4. Appetite Assessment

To assess appetite sensation, visual analogue scales (VASs) were used. These were all 100 mm in length and anchored with words at each end, expressing the most positive and the most negative rating. Hunger, satiety, fullness, and prospective food consumption were assessed. Questions were asked as follows: (1) How hungry do you feel? (2) How satisfied do you feel? (3) How full do you feel? (4) How much do you think you can eat? Here, satiety is understood as between-meal satiety, which refers to the state of inhibition of eating, and fullness is defined as the sensation of the degree of stomach filling [26]. The use of VASs to assess subjective appetite sensation has been validated for its employment in postprandial single-meal studies [27].

### 2.5. Biochemical Determinations

Blood samples were centrifuged, and sera were separated to determine the levels of glucose, cholesterol, triglycerides, insulin, and HDL cholesterol. Samples that were not immediately analyzed were stored at −70 °C for later analysis. Glucose, cholesterol, triglycerides, and HDL cholesterol analyses were performed using the Architect Clinical Chemistry Autoanalyzer System (Abbott Laboratories, Chicago, IL, USA). Insulin was measured using chemiluminescent microparticle immunoassay. Insulin assay imprecision was <7% of the total coefficient of variation. All samples were batch analyzed by the same researcher within a single assay at the end of the study in order to eliminate interassay variability.

### 2.6. Statistical Analysis

A total of 13 participants were estimated in the study in order to obtain a power of 0.8 to detect a difference of 30% between supplements on our primary variable plasma triglycerides. The anticipated dropout rate was set to 30%. To compare the results of VAS scores between treatments, the data were expressed as absolute changes (mm VAS) from the baseline (0 min). Data are expressed as mean ± standard error of the mean (SEM), unless otherwise specified. The D’Agostino-Pearson normality test was performed to assess whether the data were consistent with the Gaussian distribution. Increase in the concentration of postprandial triglycerides (TG) at a determined time point (Δ-TG) was calculated by subtracting the concentration at time 0 from the concentration at other time points. Repeated-measures analysis of variance (ANOVA) of two factors and Tukey post-hoc test were used to evaluate the effect of the treatments, time, and the interaction between the treatments and time. One-way ANOVA with Tukey post-hoc test was employed to compare the energy intake of the same participants under different treatments. Differences were considered statistically significant at *p* < 0.05. Data were processed and analyzed using GraphPad Prism (version 7.00) statistical software (GraphPad Software Inc., San Diego, CA, USA).

## 3. Results

### 3.1. Characteristics of Participants

Total of 22 subjects were eventually included to take part in this study. However, four volunteers did not start the study; two due to personal issues and the other two because of work time commitments. Three subjects were removed for protocol violations involving alcohol consumption or intense exercise prior to the test days. One participant was unable to provide blood samples. The anthropometric and biochemical characteristics of 14 participants who completed the study are shown in Table 1. Most participants exhibited obesity (8 subjects, 57%) according to the World Health Organization (WHO) classification and 92.8% (13 subjects) had raised waist circumference (men ≥90 cm; women ≥80 cm) [28]. Also, the majority of them (57%, 8 subjects) had fasting triglyceride levels that were over 150 mg/dL and were considered to have dyslipidemia, according to the WHO classification [29].

### 3.2. Postprandial Responses

**Figure 2:**
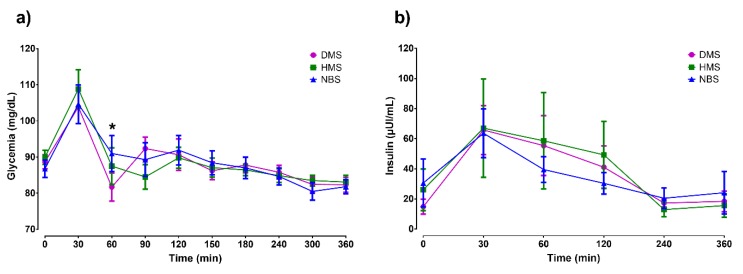
Postprandial responses of **(a)** glycemia and **(b)** insulin. The basal concentrations and the concentrations following ingestion of breakfast and treatments are shown. Data are expressed as mean ± SEM (*n* = 14). Comparisons are based on two-way repeated-measures ANOVA and the Tukey post-hoc test. * *p* < 0.01, NBS vs. DMS.

**Figure 3:**
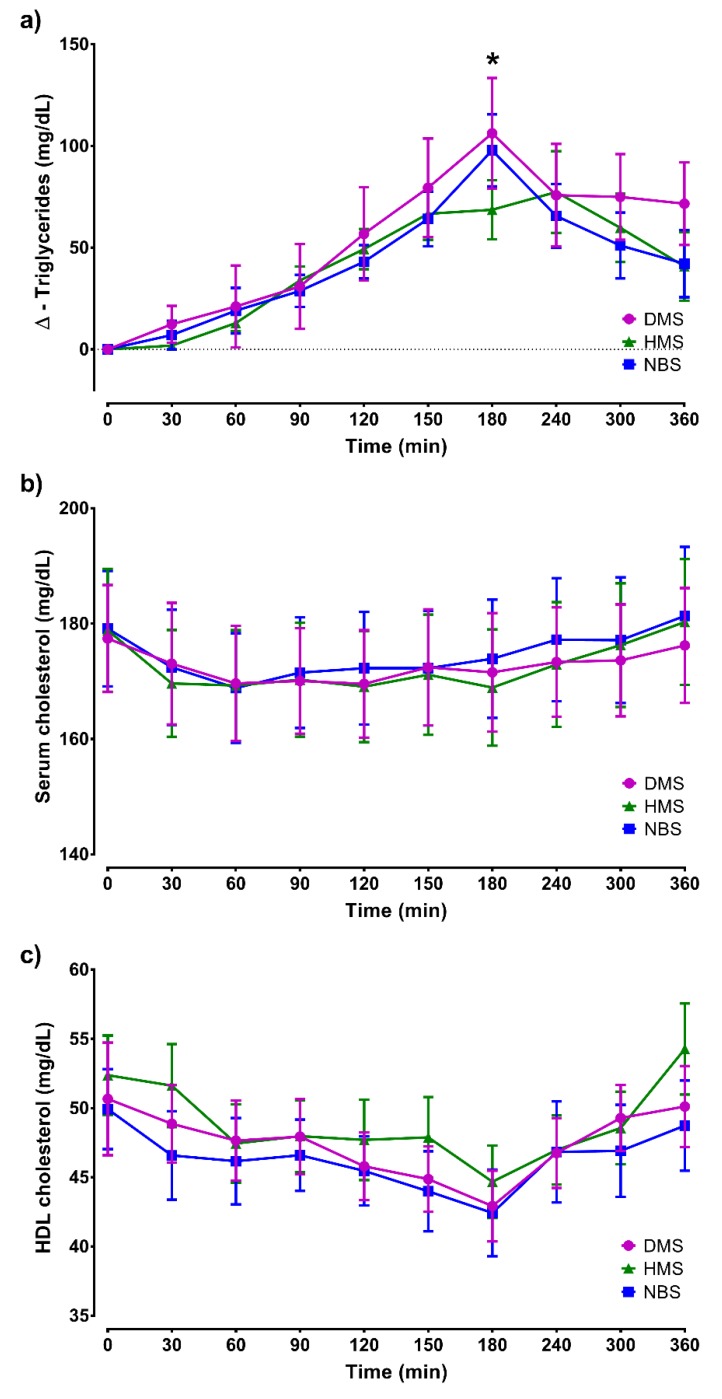
Postprandial lipemic responses: **(a)** triglycerides, **(b)** cholesterol, and **(c)** HDL cholesterol. Basal concentrations and following ingestion of breakfast and treatments are shown. Data are expressed as mean ± SEM (*n* = 14). The triglyceride values are expressed as Δ-TG. Comparisons are based on two-way repeated-measures ANOVA and the Tukey post-hoc test. * *p* < 0.05, HMS vs. DMS.

There were no significant differences in the glycemic response among treatments (*p* = 0.979) and in the interaction between time and treatments (*p* = 0.083). However, a significant difference was observed in the time factor (*p* < 0.0001). A reduction in glycemic response was observed after DMS ingestion at 60 min (NBS vs. DMS, *p* < 0.01), although the insulin response was not modified after treatments (*p* = 0.629) (Figure 2). The effect of time on triglyceride response was statistically significant (*p* < 0.001), although the treatments had no effect (Figure 3a). However, at 180 min, a reduction in the triglyceride levels was observed in the HMS group in comparison with DMS group (*p* < 0.05). No statistical differences among treatments were observed in the postprandial cholesterol or HDL cholesterol levels (Figure 3b,c).

### 3.3. Subjective Appetite Measures

**Figure 4:**
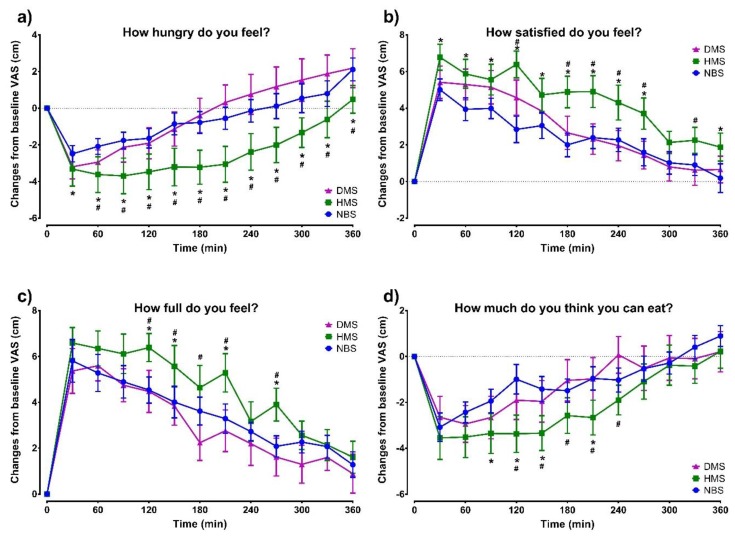
Effects of NBS on the subjective estimation of appetite using the visual analogue scale (VAS). **(a)** Hunger; **(b)** satiety; **(c)** fullness; **(d)** prospective consumption. Data are presented as changes from baseline and are expressed as mean ± SEM (*n* = 14). Comparisons are based on two-way ANOVA with the Tukey post-hoc test. * *p* < 0.05, HMS vs. NBS; # *p* < 0.05, HMS vs. DMS.

The VAS measurements are illustrated in Figure 4. Hunger sensation was significantly lower after HMS than after DMS and NBS ingestion (*F* = 2.32, *p* = 0.0006 for time × treatment interaction) from the 60 min time point until the end of the test. Satiety sensation was significantly higher after HMS than after DMS (*F* = 3.77, *p* = 0.036 for treatment effect; *F* = 1.76, *p* = 0.017 for interaction time × treatment). No significant differences were found for fullness (treatment effect: *F* = 2.70, *p* = 0.860, interaction: *F* = 1.05, *p* = 0.404). However, fullness was significantly higher after HMS, particularly from 120 to 210 min compared to DMS and NBS (*p* < 0.05). Prospective consumption was significantly lower after HMS, compared to DMS and NBS (Treatment effect: *F* = 3.66, *p* = 0.040, Interaction time × treatment *F* = 1.24, *p* = 0.208). In particular, it was significantly lower from 90 to 240 min (*p* < 0.05). Statistically significant effects of time on hunger, satiety, fullness, and prospective consumption were observed (*p* < 0.0001 in all cases).

### 3.4. Energy Intake

**Figure 5:**
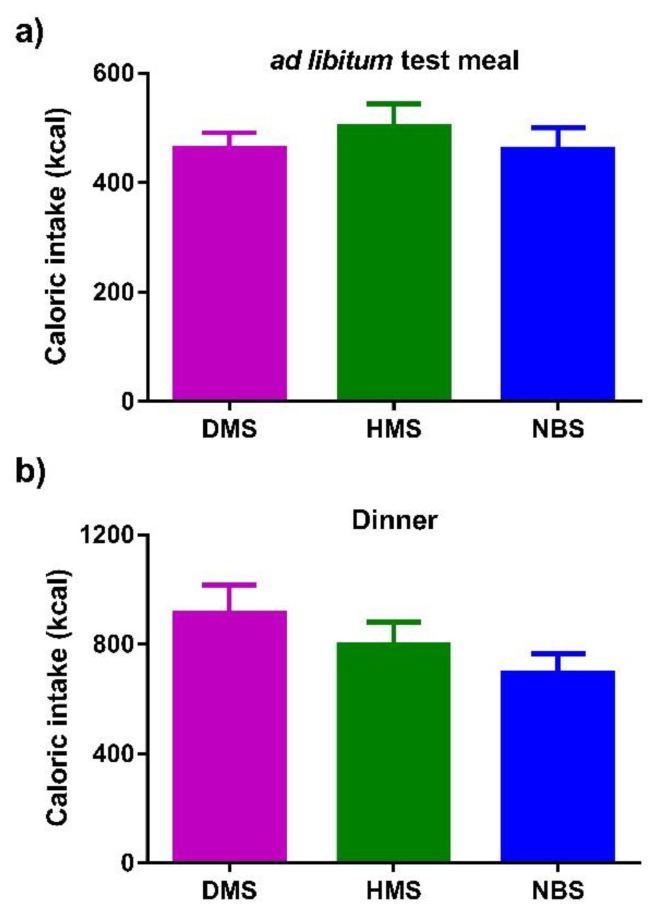
Caloric intake during the **(a)** ad libitum test meal and **(b)** dinner. Data for dinner were obtained from food records. Data are expressed as mean ± SEM (*n* = 14). Comparisons are based on one-way ANOVA with Tukey post-hoc test.

No significant differences were found in caloric intake during the ad libitum test meal (*p* = 0.260) or in caloric intake during dinner (*p* = 0.078) (Figure 5).

## 4. Discussion

The aim of this study was to compare the effects of the acute supplementation with RS from banana or maize on the postprandial lipemia, appetite sensation, and caloric intake in a group of young subjects who are overweight or obese.

With regard to glucose metabolism, in this study, acute supplementation with NBS or HMS were not observed to significantly affect postprandial glycemia or insulin. This result was expected, because all the supplements were matched for their available starch content. In previous acute studies where supplements were matched only by starch weight, the administration of RS was found to typically induce a reduction on the glycemic and insulinemic responses. These effects have been observed after providing 40 g of NBS, 50 g of raw potato starch, or 25 g of RS to healthy subjects [30,31,32]. In these experiments, the effects of RS on glycemia and insulin probably reflected the greater availability of digestible carbohydrates in the control group. However, it is difficult to compare the results of the present study with those from other groups because we used a high-fat breakfast and a longer period of study (6 h MTT) than most other studies, which used a 3 h postprandial period or a 2 h oral glucose tolerance test. In chronic studies, however, in which RS is administered in the long term, the beneficial effect of RS in improving insulin sensitivity have been consistently demonstrated [16,33,34,35,36].

Based on the results of the present study, we were not able to prove our hypothesis that supplementation with RS improves postprandial lipemia. As a nonviscous, highly fermentable fiber, it was assumed that RS would act through one of the several mechanisms known, for example, slowing of gastric emptying, reduction in TG hydrolysis, inhibition of pancreatic lipase activity, alterations in micelle formation, and modulation of the intestinal secretion of chylomicrons [37]. However, no effects on postprandial lipemia were observed after both HMS or NBS supplementation. To date, there is a paucity of studies analyzing the effects of acute RS supplementation on postprandial lipemia [20,21]. In one such acute intervention where healthy subjects received a meal supplemented with 30 g raw potato starch containing 19.5 g of RS, postprandial triglycerides, and cholesterol were not modified, although a reduction in triglycerides-rich lipoproteins was observed [20]. In a more recent study, 5 or 10 g of resistant maltodextrin induced a reduction in postprandial triglycerides and insulin in healthy subjects [21]. The differences in the results of these studies may be partially explained by the distinct physicochemical properties of the used substances. That is, raw potato starch is considered as a type 2 resistant starch that is similar to NBS and HMS and is protected from digestion because of its crystalline structure. In contrast, resistant maltodextrin is considered a type 5 starch containing 1–2 and 1–3 glycosidic linkages [7].

With regard to chronic supplementation of RS in humans, most studies have not found a reduction on postprandial lipemia. For example, when RS from high-amylose maize was provided at a dose of 40 g/d for 8 or 12 weeks to subjects with insulin resistance, no effects on postprandial lipemia were observed [17]. Additionally, when a 4-week diet rich in arabinoxylan (16 g/d) and RS (21 g/d) was administered to patients with metabolic syndrome, no effects were found [18]. These, and other reports in which no effect of RS supplementation on postprandial lipemia was observed in humans [19,38], appear to contrast findings from animal models wherein a reduction in postprandial lipemia was frequently observed [10,12,13,39,40,41,42,43,44,45].

The present results showed that all measures of subjective appetite were positively affected by the HMS supplement but not by the NBS supplement, even though the quantity of RS in these supplements was the same (20 g) and both of them are classified as type 2 starches. Differences in structure that determine the properties such as crystallinity, amylose to amylopectin ratio, and granular structure could have influenced these different effects on appetite sensations. Despite this, it is difficult to explain these findings. In a previous study from our group, acute NBS supplementation did not affect appetite sensation, even though a reduction in the ad libitum caloric intake was observed at the time of the test meal [30]. However, comparison between these studies should be done carefully; in that study, the supplements were dissolved in water and not matched for available carbohydrates. Also, no high-fat breakfast was provided to the participants and the MTT lasted only 3 h. On the other hand, the inconsistency between appetite sensation scores and energy intake deserves further elucidation.

This study has several strengths. First, this is to our knowledge the first study to investigate the effects of two type 2 resistant starches from different sources on postprandial lipemia and appetite. Second, the crossover design reduces the influence of confounding covariates. However, several limitations are also present. First, the study group included more female subjects, so the results may not be representative of the male population. Second, the study sample was relatively small. Although, a sample size calculation was performed, it is possible that a larger sample size may have resulted in a different outcome. Third, Apo B48 and gastrointestinal peptides were not assessed.

## 5. Conclusions

In conclusion, the findings of the present study indicate that a single acute dose of RS cannot improve postprandial lipemia in subjects who are overweight or obese on a high-fat meal; yet, the potential benefits of long-term supplementation should not be ruled out based on these results. In addition, HMS had significant positive effects on subjective appetite sensations, but there was no associated effect on the ad libitum caloric intake.

## Figures and Tables

**Table 1:** Baseline characteristics of the study participants.

Characteristic	Female	Male	Total
Subjects (*n*)	11	3	14
Age (year)	21.18 ± 0.76	21.67 ± 1.67	21.29 ± 0.67
Height (cm)	156.50 ± 1.12	175.30 ± 3.84	160.50 ± 9.06
Body weight (kg)	75.55 ± 3.80	98.17 ± 7.85	80.39 ± 4.17
BMI (kg/m^2)^	30.77 ± 1.29	31.80 ± 1.21	30.99 ± 1.03
Fat percentage (%)	36.39 ± 2.12	27.50 ± 1.80	34.49 ± 1.96
SBP (mm Hg)	119.50 ± 3.23	128.30 ± 11.14	121.40 ± 3.37
DBP (mm Hg)	73.64 ± 2.55	73.33 ± 4.10	73.57 ± 2.12
Waist (cm)	100.20 ± 8.13	104.00 ± 1.53	101.00 ± 6.34
Hip (cm)	109.10 ± 2.64	115.80 ± 4.32	110.60 ± 2.32
Waist to Hip Ratio	0.93 ± 0.09	0.90 ± 0.00	0.92 ± 0.07
**Fasting concentrations:**			
Glucose (mg/dL)	84.27 ± 1.94	89.00 ± 5.20	86.00 ± 1.84
Insulin (µUI/mL)	5.30 (3.62, 9.13)	9.66 (5.54, 38.70)	5.72 (3.86, 10.45)
Triglycerides (mg/dL)	154.0 (91.00, 238.00)	282 (98.00, 441.00)	163.00 (96.25, 249.00)
Total cholesterol (mg/dL)	184.5 ± 11.59	217.7 ± 27.30	185.90 ± 9.15
HDL cholesterol (mg/dL)	48.57 ± 4.03	38.70 ± 6.91	48.22 ± 3.64

Data are expressed as mean ± standard error of the mean (SEM) or median (25th and 75th percentiles). BMI, body mass index; DBP, diastolic blood pressure; HDL, high-density lipoprotein; SBP, systolic blood pressure.
